# Supplementary material for: ProteinShader: illustrative rendering of macromolecules
Source: BMC Struct Biol. 2009 Mar 30;9:19. doi: 10.1186/1472-6807-9-19 (PMC2672931; doi:10.1186/1472-6807-9-19)
Supplement: Additional file 1 — ProteinShader program without source code. This compressed file contains the complete ProteinShader program including associated libraries, but no source code. A README.txt file gives an overview of the ProteinShader distribution, and the index.html file in the help subdirectory has directions on getting started with the program as well as a set of tutorials. [file 1472-6807-9-19-S1.zip › ProteinShader-beta-0_9_4-binary/help/api/org/proteinshader/gui/enums/class-use/OrientationEnum.html]

Uses of Class org.proteinshader.gui.enums.OrientationEnum (ProteinShader API)


|  |  |  |
| --- | --- | --- |
| **Overview** \| **Package** \| **Class** \| **Use** \| **Tree** \| **Deprecated** \| **Index** \| **Help** | |  |
| PREV   NEXT | **FRAMES**    **NO FRAMES**     **All Classes** |  |


---


## **Uses of Class org.proteinshader.gui.enums.OrientationEnum**

| Packages that use OrientationEnum | |
| --- | --- |
| **org.proteinshader.gui** | Holds all of the Swing GUI components and their associated listeners, including class Renderer, which is registered as a listener for the GLCanvas object that is used a drawing surface. |
| **org.proteinshader.gui.components** | Holds the gui components (JPanels and JMenus) needed by class ProteinShaderGUI. |
| **org.proteinshader.gui.enums** | Holds the enums needed by org.proteinshader.gui and its subpackages. |
| **org.proteinshader.gui.viewing** | Holds important helper classes that the Renderer uses to control the view: Camera, Rotation, Lighting, *etc*. |

Uses of OrientationEnum in org.proteinshader.gui

| Methods in org.proteinshader.gui with parameters of type OrientationEnum | |
| --- | --- |
| `void` | `MediatorImpl.setImageOrientation(OrientationEnum orientation)`             Sets the image to the requested orientation (Front, Back, Left, *etc*.). |
| `void` | `Renderer.setImageOrientation(OrientationEnum orientation)`             Resets the orientation of the image by resetting the angle of rotation about the x-axis and the angle of rotation about the y-axis. |
| `void` | `Mediator.setImageOrientation(OrientationEnum orientation)`             Sets the image to the requested orientation (Front, Back, Left, *etc*.). |

Uses of OrientationEnum in org.proteinshader.gui.components

Constructors in org.proteinshader.gui.components with parameters of type OrientationEnum

`SpringLoadedJFrame(JFrame parent,
OrientationEnum position)`
  
          Constructs a SpringLoadedJFrame using default width and height
factors based on whether the spring-loaded frame is attached to
its parent frame at the TOP, BOTTOM, LEFT, or RIGHT (see comments
for DEFAULT\_SHORT\_FACTOR and DEFAULT\_LONG\_FACTOR).

`SpringLoadedJFrame(JFrame parent,
OrientationEnum position,
double widthFactor,
double heightFactor)`
  
          Constructs a SpringLoadedJFrame.

Uses of OrientationEnum in org.proteinshader.gui.enums

| Methods in org.proteinshader.gui.enums that return OrientationEnum | |
| --- | --- |
| `static OrientationEnum` | `OrientationEnum.valueOf(String name)`             Returns the enum constant of this type with the specified name. |
| `static OrientationEnum` | `OrientationEnum.valueOfMenuName(String menuName)`             Returns the OrientationEnum with the same menu name as the String given as an argument. |
| `static OrientationEnum[]` | `OrientationEnum.values()`             Returns an array containing the constants of this enum type, in the order they're declared. |

Uses of OrientationEnum in org.proteinshader.gui.viewing

| Methods in org.proteinshader.gui.viewing with parameters of type OrientationEnum | |
| --- | --- |
| `void` | `Rotation.setImageOrientation(OrientationEnum orientation)`             Resets the orientation of the image by resetting the angle of rotation about the x-axis and the angle of rotation about the y-axis. |

---


|  |  |  |
| --- | --- | --- |
| **Overview** \| **Package** \| **Class** \| **Use** \| **Tree** \| **Deprecated** \| **Index** \| **Help** | |  |
| PREV   NEXT | **FRAMES**    **NO FRAMES**     **All Classes** |  |


---

# *Copyright © 2007-2008*
